# A novel method for irrigating plants, tracking water use, and imposing water deficits in controlled environments

**DOI:** 10.3389/fpls.2023.1201102

**Published:** 2023-08-29

**Authors:** Alex Cichello, Austin Bruch, Hugh J. Earl

**Affiliations:** Department of Plant Agriculture, University of Guelph, Guelph, ON, Canada

**Keywords:** irrigation, drought stress, drought tolerance, water use, water table, high-throughput phenotyping, physiological maturity, controlled environment

## Abstract

The study of genomic control of drought tolerance in crops requires techniques to impose well defined and consistent levels of drought stress and efficiently measure single-plant water use for hundreds of experimental units over timescales of several months. Traditional gravimetric methods are extremely labor intensive or require expensive technology, and are subject to other errors. This study demonstrates a low-cost, passive, bottom-watered system that is easily scaled for high-throughput phenotyping. The soil water content in the pots is controlled by altering the water table height in an underlying wicking bed via a float valve. The resulting soil moisture profile is then maintained passively as water withdrawn by the plant is replaced by upward movement of water from the wicking bed, which is fed from a reservoir via the float valve. The single-plant water use can be directly measured over time intervals from one to several days by observing the water level in the reservoir. Using this method, four different drought stress levels were induced in pots containing soybean (*Glycine max* (L.) Merr.), producing four statistically distinct groups for shoot dry weight and seed yield, as well as clear treatment effects for other relevant parameters, including root:shoot dry weight ratio, pod number, cumulative water use, and water use efficiency. This system has a broad range of applications, and should increase feasibility of high-throughput phenotyping efforts for plant drought tolerance traits.

## 1 Introduction

Soil water deficits constitute one of the major limitations to productivity of field crops in North America, and yield losses due to meteorological drought effects are increasing over time in some regions of the continent (Li et al., 2009; Zipper et al., 2016). Current changes in climate are unprecedented over a timescale of many centuries, and there is increasing evidence that human activities are contributing to increasing frequency of agricultural droughts (Shukla et al., 2019; Masson-Delmotte et al., 2021). Modelling studies have predicted that in the US Midwest, drought will be the predominant cause of soybean yield reductions due to climate change by the middle of the 21st century (Jin et al., 2017). The reductions in yield loss predicted by these studies typically do not account for genetic improvement of crops over time, which could reduce yield losses with targeted breeding programs.

Reducing soybean yield losses from drought stress may require the development of novel, drought-tolerant cultivars. These breeding efforts will benefit from identification of loci within the soybean genome that contribute to drought tolerance, defined here as maintenance of a high fraction of potential seed yield when soil water availability is limiting to growth and seed set. Modern tools such as Genome Wide Association Studies (GWAS) are effective means of associating particular loci with variation for growth and yield traits in many crops, including soybean. This approach requires the phenotyping of large diversity panels for which genotypic data are available (Huang and Han, 2014). The completion of the reference genome for soybean, as well as the advent of high-throughput genotyping methods (e.g. via genotype-by sequencing), have enabled GWAS for dissecting genetic control of quantitative traits (Huang and Han, 2014). However, because GWAS requires the phenotyping of hundreds of lines, the phenotypic screening component can create logistical and technical challenges, depending on the traits of interest. This scale of data collection is practically performed for certain agronomic traits that are routinely measured in breeding programs (e.g. yield, lodging scores, plant height, days to maturity, seed oil and protein from NIR measurements) using small plots in the field (Spindel et al., 2018; Ravelombola et al., 2021), and so GWAS has been applied to investigating genetic control of these traits in dozens of studies including thousands of different accessions (Shook et al., 2021). Ground and air-based remote sensing methods have also been developed to measure canopy traits in a high-throughput fashion (Bai et al., 2016; Han et al., 2018; Spindel et al., 2018; Hong et al., 2019).

Phenotypic data for water use traits would greatly benefit GWAS for drought tolerance in crops like soybean. However, high-throughput phenotyping for water use traits is impractical in field plots, so phenotyping is often done under controlled environment conditions instead. Unfortunately, controlled-environment methods for imposing defined levels of water stress and measuring water use tend to be technology- or labor-intensive on a per-entry basis, or risk damaging the plants (Earl, 2003; Granier et al., 2005; Gebre and Earl, 2020; Gebre and Earl, 2021; Joshi et al., 2021; Liyanage et al., 2022). Further, gravimetric methods usually fail to account for increasing plant biomass, resulting in progressive under-replacement of water to the pot and a drying of the soil over time.

As a result of these technical challenges, most controlled environment high-throughput phenotypic screenings of traits related to drought stress have concentrated on easily evaluated traits, measured on seedlings or seeds (Zeng et al., 2017; Khan et al., 2018; Khan et al., 2019; Yu et al., 2019; Liu et al., 2020; Sharmin et al., 2021; Rahimi et al., 2022). These traits may not be useful for assessing genetic control of drought effects on seed yield and yield components, which are expressed much later in the developmental cycle (Doss et al., 1974; Pandey et al., 1984; Desclaux et al., 2000; Zatybekov et al., 2017). Future phenotyping studies should measure effects of stress on yield components, such as pod number and seeds per pod, as well as water use and water use efficiency during critical developmental phases (Hufstetler et al., 2007). Therefore, there remains a need for methods to apply well defined levels of water stress and measure water use on hundreds of experimental units through reproductive development right to maturity.

We demonstrate the function of a bottom-watered, gravity-fed system which can maintain precise control of soil moisture within a rooting column, throughout the entire soybean growth cycle. The basis of this system was originally designed by Bruch (2020). This system wicks water upwards from a pot-specific water table in a lower wicking bed into an upper rooting column using a hydraulically conductive mix of granitic sand and Turface. The water table height in the wicking bed can be adjusted as required to alter the soil moisture profile in the upper rooting column in a predictable fashion. After a brief re-equilibration period, the pot maintains the new soil moisture profile for the duration of the growth cycle, even as the plant draws water from the rooting column. The water-level adjustment reduces the individual plant water use in a predictable fashion (Bruch, 2020). The system contains few moving parts and no electronic components, and thus has a low per-unit cost to build. The flow ports through which water is fed to the pots can be connected in parallel via manifolds, making it possible to control several pots at once with a single float valve. While this design makes the current system easily adaptable to high-throughput applications for phenotypic screenings of large populations, the sand and Turface mix used by Bruch (2020) could not grow healthy soybean plants to maturity in this system – plants developed brown, necrotic leaf spots and often died well before maturity (Cichello, 2023). The present study therefore replaced Turface with an alternative calcined clay material to maintain the pot hydrology observed by Bruch (2020), while also supporting healthy plant growth to physiological maturity so that the effects of the water stress treatments on final seed yield and yield components could be determined.

The system presented here fundamentally differs from the techniques presented in Haan and Barfield, 1971 and Snow and Tingey, 1985. Specifically, the aforementioned systems focus on keeping roots confined largely to a 2-dimentional interface at a set distance above a water column maintained by porous media underlying the soil. In contrast, the wicking action of the current system relies on a specialized potting mix with a soil-specific water release curve to achieve the drought effect, thereby maintaining a stable soil moisture profile throughout the whole pot. This adaptation expands the soil volume available to support plant growth, allowing for unrestricted root growth through the hydraulically conductive medium itself and preventing the unrealistic build-up of roots at the interface of the soil and conductive media observed in earlier systems (Snow and Tingey, 1985). Further, by maintaining a stable soil moisture profile via upward wicking from the water table into the vadose zone, the current system improves upon the method proposed by Iseki et al. (2018), allowing for valid water use measurements and consistent drought intensities over the duration of the growth cycle. In this study, the effect of water table depth below the rooting column on soil moisture profile and water use for individual plants using this system were investigated. Yield and growth traits were also measured to test the effect of the soil moisture level on growth and traits which would be of interest to soybean breeding programs for the development of new, drought-tolerant cultivars.

## 2 Materials and methods

### 2.1 Growth environment

Plants were grown on a reinforced bench in a Growth Room at the University of Guelph. The growth room is maintained at a temperature of 23°C during the day and 21°C at night at a relative humidity of 70%. The incident PAR ranged from ~230 µmol m^-2^ s^-1^ at the soil surface to ~300 µmol m^-2^ s^-1^ at the approximate maximum height of the plants during the growth cycle, using GE 93140 broad-spectrum white LED lamps (GE Lighting, East Cleveland, OH, United States). The photoperiod was maintained at 16 hours of daylight and 8 hours of darkness.

### 2.2 Growth system

Plants were grown in a passive-flow, bottom-watered system designed to maintain a constant, operator-controlled soil moisture profile (Bruch, 2020). For the current experiment, each pot was attached to its own reservoir apparatus comprising a 75-cm tall, 5.1-cm ID ABS pipe positioned vertically and capped at the bottom with a plastic piping cap. A push-fitting elbow joint was installed on each reservoir a height of approximately 1.25 cm from the bottom to allow for the attachment of a 3-mm ID plastic hose to facilitate water flow towards the float valve and then the pot.

**Figure 1:**
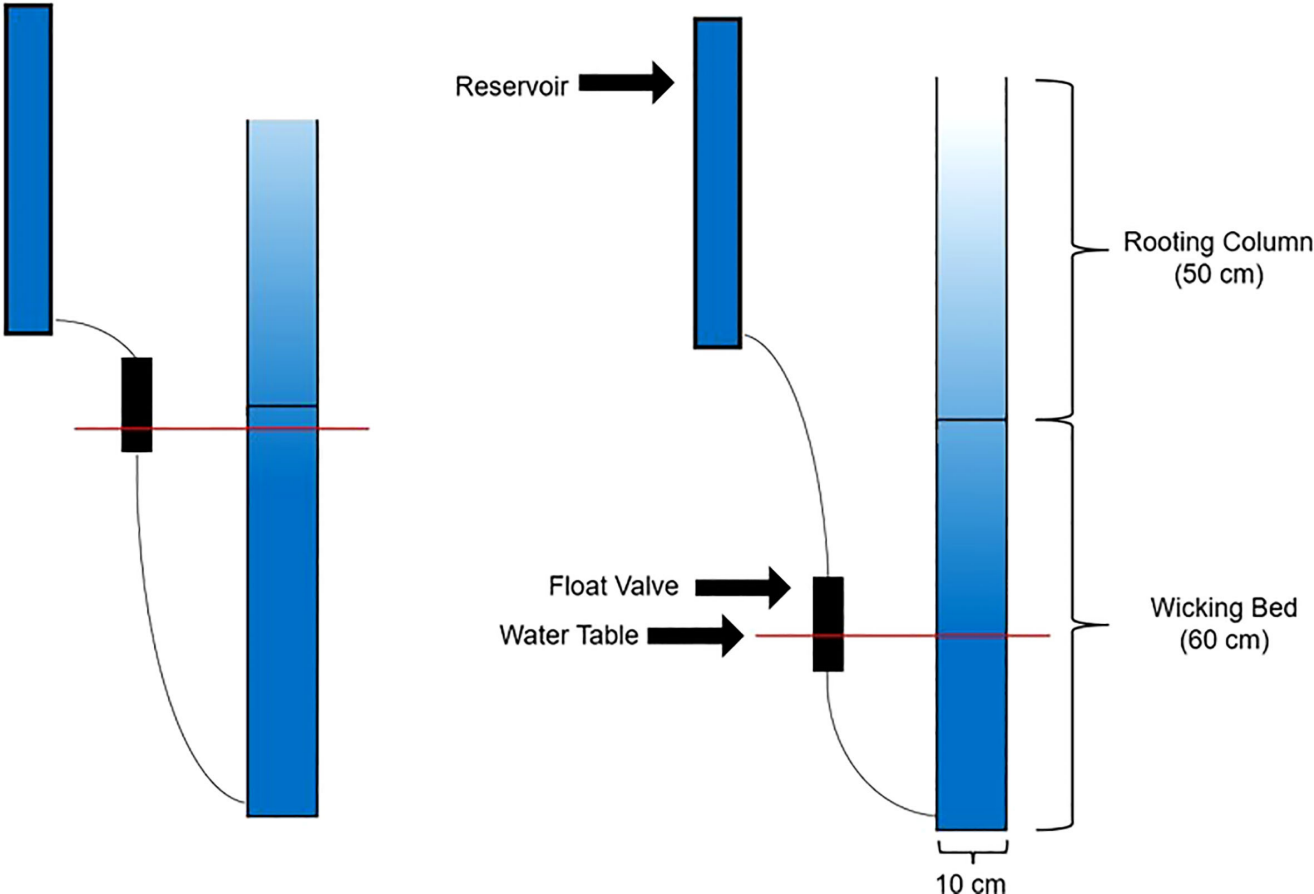
Schematic of the drought system designed by Bruch (2020). The pot is divided into an upper rooting column and a lower wicking bed. Water is stored in the reservoir and fed to the bottom of the wicking bed on an as-needed basis, through the action of a float valve. The float valve sets the pot-specific water table level and can be physically raised and lowered to change the soil moisture profile of the rooting column.

The pot apparatus is described in **Figure 1**. Each pot consists of two components. The lower section of the pot is a wicking bed and the upper portion is a rooting column. Both the wicking bed and the rooting column are filled with the soil mix, which consists of 60% (by volume) granitic sand (B-sand, Hutcheson Sand and Mixes, Huntsville, ON, Canada) and 40% fired calcine clay (Safe-T-Sorb, Toronto Salt & Chemicals, Brampton, ON, Canada). This soil mix was previously shown to provide a soil moisture profile within the rooting column that was strongly dependent on the water table height in the underling wicking bed (Cichello, 2023). The wicking bed is a 60 cm tall PVC tube (10 cm ID), fitted at the bottom with a PVC cap. About 2.5 cm from the bottom, each wicking bed was fitted with a hose barb to connect it to the float valve via a plastic hose, and an elbow joint, to which a piece of 3.2 mm translucent poly tubing (Rubberline Products Ltd., Kitchener, ON, Canada) was attached and oriented vertically to serve as a sight glass, to permit observation of the water table height within the wicking bed. Once the wicking bed was filled with soil, a piece of 36-µm, Nitex nylon mesh (Dynamic Aqua Supply, Surrey, BC, Canada) was secured across the top of the wicking bed using an elastic band. This nylon membrane allows water from the wicking bed to wick upwards to the rooting column, while preventing the roots in the rooting column from penetrating down into the wicking bed. The upper rooting column consists of a 50-cm section of PVC tube. The 50-cm height was initially chosen during the preliminary work for this study, based on a soil medium of sand and Turface (Bruch, 2020). In the present work, Turface was replaced with the Safe-T-Sorb as a component of the growth medium since preliminary studies showed that, unlike Turface, it could support healthy plant growth up to physiological maturity (Cichello, 2023).

Soil Moisture Measurement (SMM) probe ports were drilled into the rooting column at distances of 10 cm, 20 cm, 30 cm and 40 cm from the bottom of the rooting column. These probe ports each consisted of two 5-mm diameter holes 3.2 cm apart to facilitate Time Domain Reflectometry (TDR) measurements of volumetric soil water content (VSWC). Rooting columns were lined internally with polyethylene plastic sleeves to permit easy removal of the intact soil columns and root systems at the end of the experiment. Each wicking bed was fixed to one rooting column using a rubber coupling to complete one pot. One 7.5-cm long 5-mm dia. stainless steel screw was then installed in each of the eight holes in the rooting column to complete the SMM ports.

**Figure 2:**
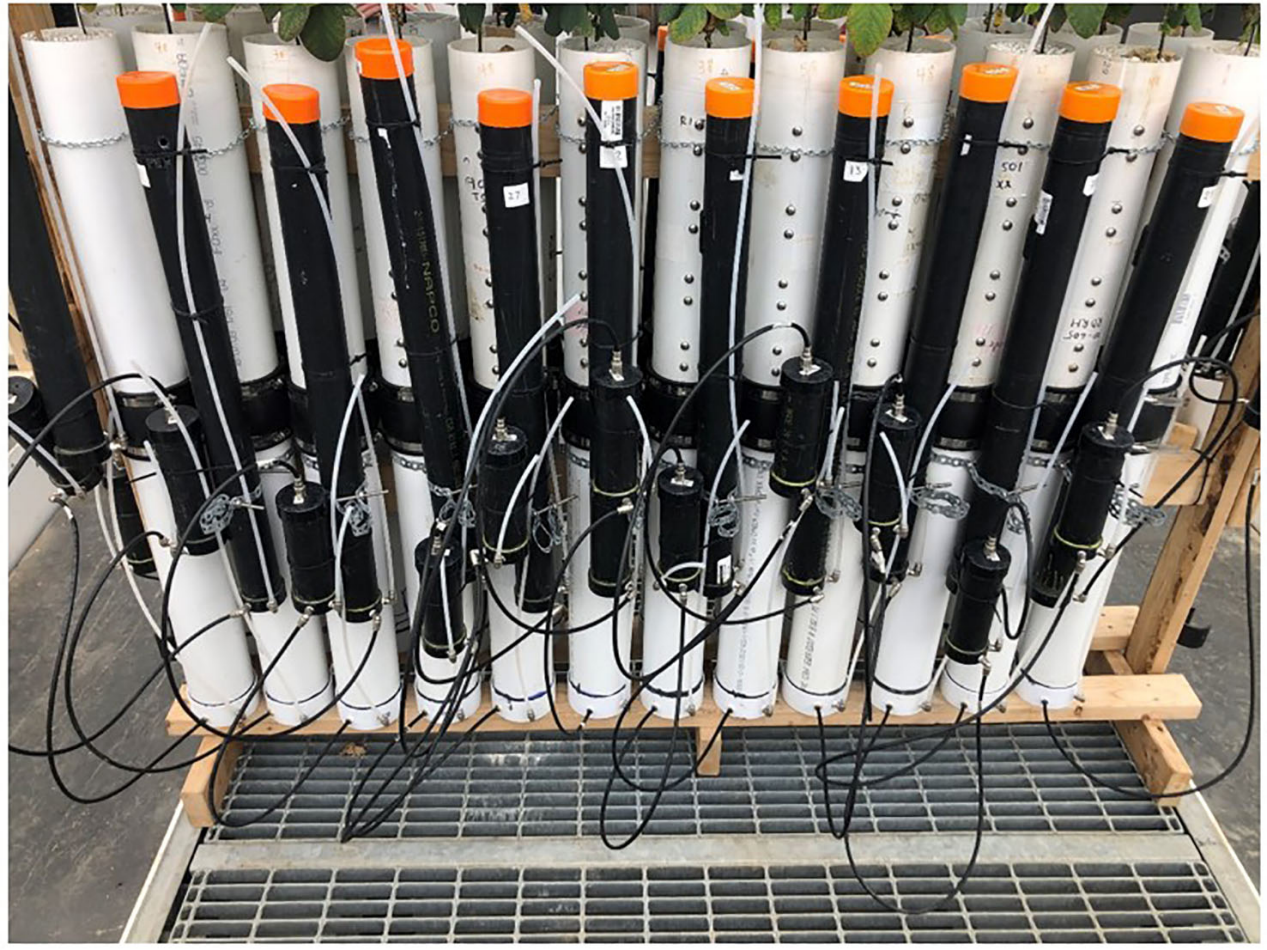
White PVC pots are divided into lower wicking beds and upper rooting columns, secured together with rubber couplings. TDR measurement ports are visible on rooting columns. Hoses connect black reservoirs to float valves, and route the water from the float valves to the flow ports on the lower wicking beds. Float valve height is adjustable to allow for regulation of soil moisture in the upper rooting column.

Each pot was fitted with an individual reservoir and each pot’s control level was independently controlled by a single float valve, constructed from a section of 51 mm ID ABS plastic pipe, capped at both ends. Water from the reservoir entered the valve body via a hose barb in the top cap. A float constructed from a pill bottle with neoprene rubber glued to its cap rose with the water level in the valve body, until the water level within the float valve was high enough that the neoprene rubber was seated against the hose barb, stopping the flow of water. The reservoir and float valve apparatus for each pot was attached directly to the pot itself (**Figure 2**).

### 2.3 Calibration curve

A calibration curve was generated for the soil medium tested to relate TDR readings to VSWC values. This curve was created by measuring the TDR for the soil mix at increasing VSWC.

The soil moisture measurements for the calibration curve were taken in a custom-made calibration tube, consisting of 10-cm PVC pipe with a 10-cm PVC cap attached to the bottom. Two screws (identical to the ones described above) were added to the PVC cap, spaced ~3.2 cm apart. The screws were in the bottom cap oriented vertically, to maximize the vertical coverage of the measurement, thereby minimizing the effects of gravimetric potential on measured VSWC.

A dry, 2245-ml sample of the soil mix was measured out into a mixing tub, which was placed on an electronic balance. A subsample of the soil mix was scooped into the calibration tube. Four TDR measurements were then taken with a TDR meter (FieldScout™, Spectrum Technologies, Aurora, IL, United States), by touching the signal terminals of the TDR meter to the heads of the screws. The soil in the calibration tube was then replaced into the mixing tub. Water was then added to the mixing tub in increments of 112.25 ml corresponding to 5% increases in VSWC. After each addition of water the soil was thoroughly mixed and another subsample added to the calibration tube. This wetting and measurement cycle was repeated in 5% increments of VSWC until the soil in the mixing tub became water-saturated.

**Figure 3:**
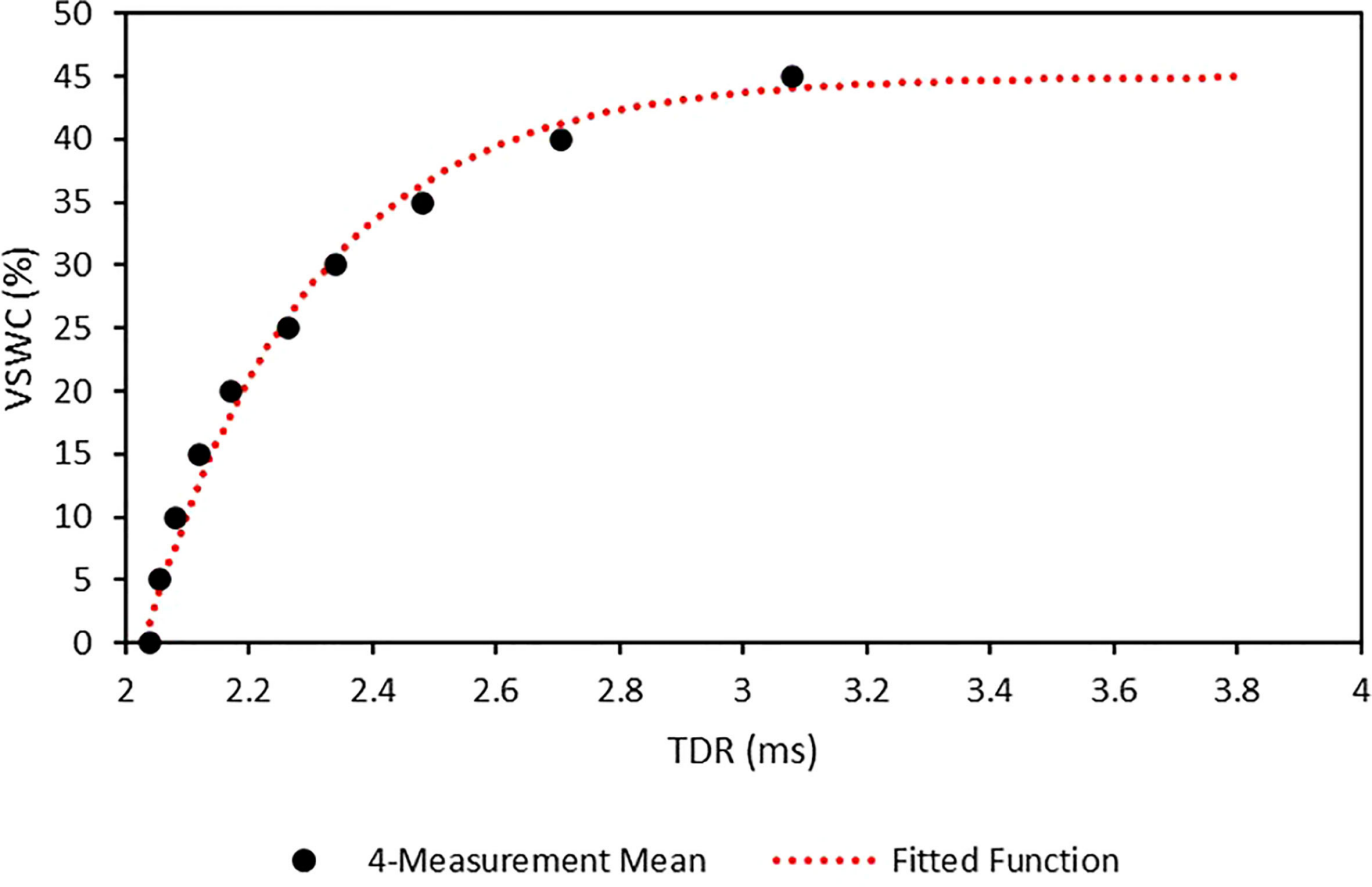
TDR calibration measurement means and fitted calibration function for the calcined clay soil mix used in this study. Points are the means of 4 measurements. Calibration function was generated using PROC NLIN in SAS v. 9.4. Standard errors for the measurement means are negligible at this scale.


The four TDR measurements for each VSWC were averaged and plotted against their corresponding VSWCs (**Figure 3**). A calibration curve was fitted using the PROC NLIN procedure in SAS v.9.4 (SAS Institute Inc., Cary, NC, United States). The fitted function was:

$$\text{VSWC}=45\left(1-e^{-3.67(\left(x/1000\right)-2.03)}\right)$$

where VSWC is the % volumetric soil water content and x is the TDR value in µs.

### 2.4 Pot preparation

Washed, inert aquarium gravel (Super Naturals, Zen Garden, CaribSea, Fort Pierce, FL, United States) was added to the bottom of each wicking bed to a depth of approximately 5 cm to allow for free flow of water into the pot via the flow port. The wicking beds were then filled with the mixed soil medium and tapped repeatedly with a rubber mallet to settle the soil mix in the wicking bed and minimize air pockets. The nylon membranes were attached to the top of each wicking bed and the rubber coupling was affixed by tightening the lower hose clamp, and then the top rooting column was placed on the completed wicking bed and attached by tightening the other hose clamp on the coupler. The rooting columns were then lined with polyethylene sleeves (Poly Tubing, Uline, Pleasant Prairie, WI, United States) and filled with the soil mix and tapped lightly with a rubber mallet to settle the soil mix in the rooting column and minimize air pockets. Finally, the stainless steel screws were screwed into the SMM ports to facilitate TDR measurements.

The pots were placed in position on a wooden rack in the growth room. The reservoirs were secured to the wicking beds using pipe strapping. The float valves were secured to the reservoirs with zip-ties, tightly enough to secure them in place but loosely enough to allow for repositioning. Float valves were positioned to control each pot-specific water table height at 2 cm below the nylon rhizobarrier at the top of the wicking bed (58 cm above the bottom of the wicking bed). Pots were then top watered to soil saturation and allowed to drain through the sight glasses so that this water table height was restored. TDR measurements of VSWC were taken prior to planting to ensure the soil moisture profiles had stabilized. The measured VSWC changed only negligibly between two and four days after top-watering, so seeds were planted four days after top-watering.

The plant material used here was *OAC Drayton*, a food-grade soybean variety of maturity group 0, developed at the University of Guelph, provided by Dr. Istvan Rajcan. Three seeds of *OAC Drayton* were planted in each pot on January 14^th^, 2022. Plastic caps were placed on each pot to minimize the effect of evaporation on the upper layers of soil. The majority of the seedlings emerged at 4 Days After Planting (DAP), so these caps were removed at 4 DAP and replaced with white aquarium gravel (Spectrastone, Estes Gravel Products, Fairfield, NJ, United States) to a depth of approximately 1 cm. Seedlings were thinned to one per pot at 6 DAP. Pots were watered with 100 ml 1% w/v 20:20:20 N:P:K + micronutrients (Plant Products, Leamington, ON, Canada) at 0 and 14 DAP. At 45 DAP, pots were again watered with 100 ml 1% w/v 20:20:20 N:P:K, but amended with 0.25% w/v MgSO_4_ and 0.25% w/v Ca(NO_3_)_2_. Fly paper was installed on two border pots on the corner of the rack at planting to act as a pest countermeasure.

### 2.5 Experimental design and treatment establishment

**Figure 4:**
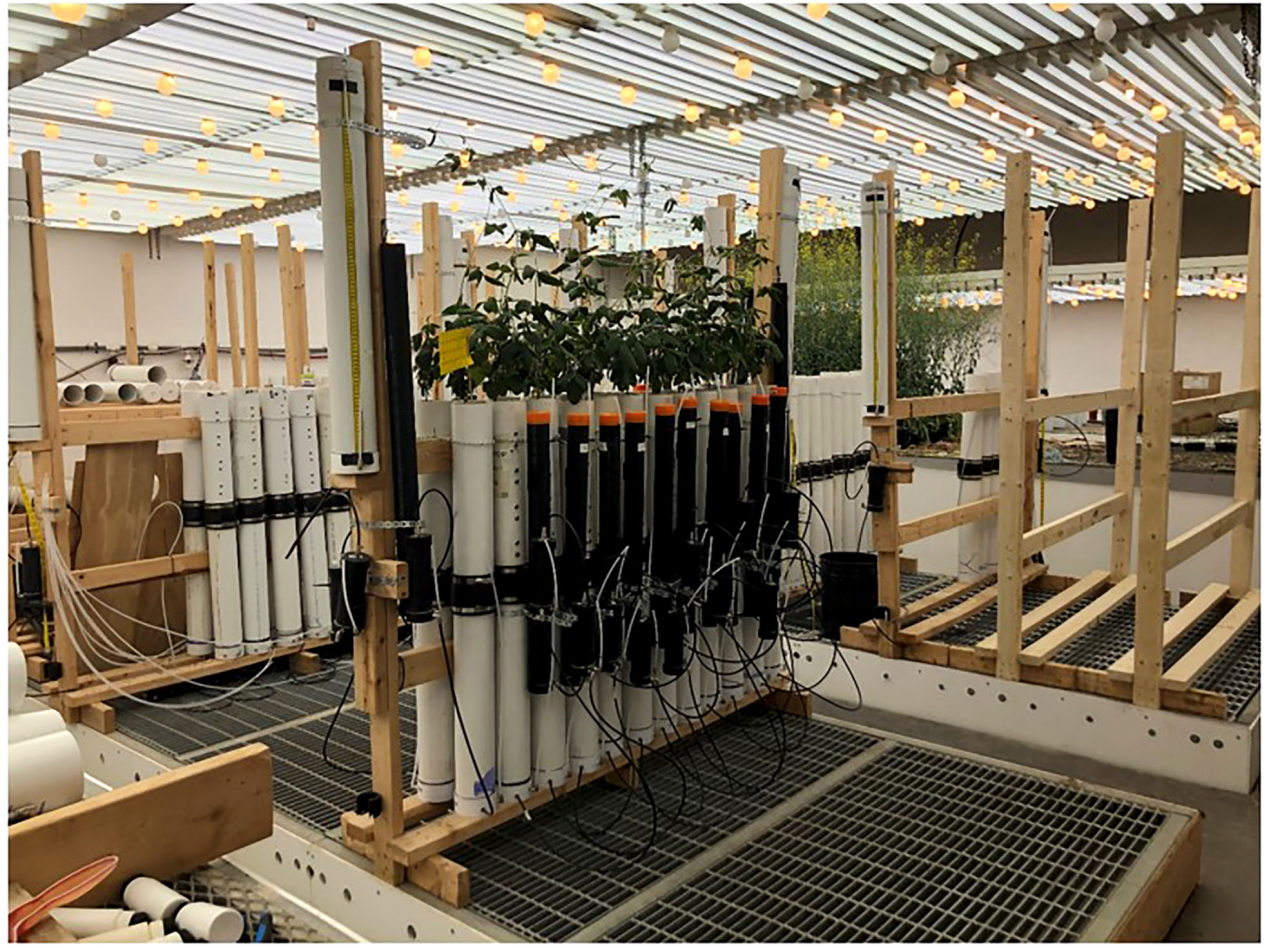
The full rack setup of the current experiment. Individual pot-reservoir systems are secured to a wooden frame in rows of two. Four corner pots act as border plants and fly paper is used as a pest countermeasure. String is tied around the frame to act as a lattice structure for plants to climb.

Pots were arranged in a Randomized Complete Block Design (RCBD) on a single rack. There were four treatments and five replications (20 experimental units) arranged in two rows of ten, plus 4 border pots (one on each end of each row. Each block comprised four pots arranged in a 2x2 square (**Figure 4**).

Initially, each pot was maintained at the control level, defined as a water table position 2 cm below the rhizobarrier. At 31 DAP, float valve heights were changed to adjust the water table height in each pot to a predetermined level according to the assigned treatment:

2 cm below rhizobarrier.

12 cm below rhizobarrier.

22 cm below rhizobarrier.

32 cm below rhizobarrier.

**Figure 5:**
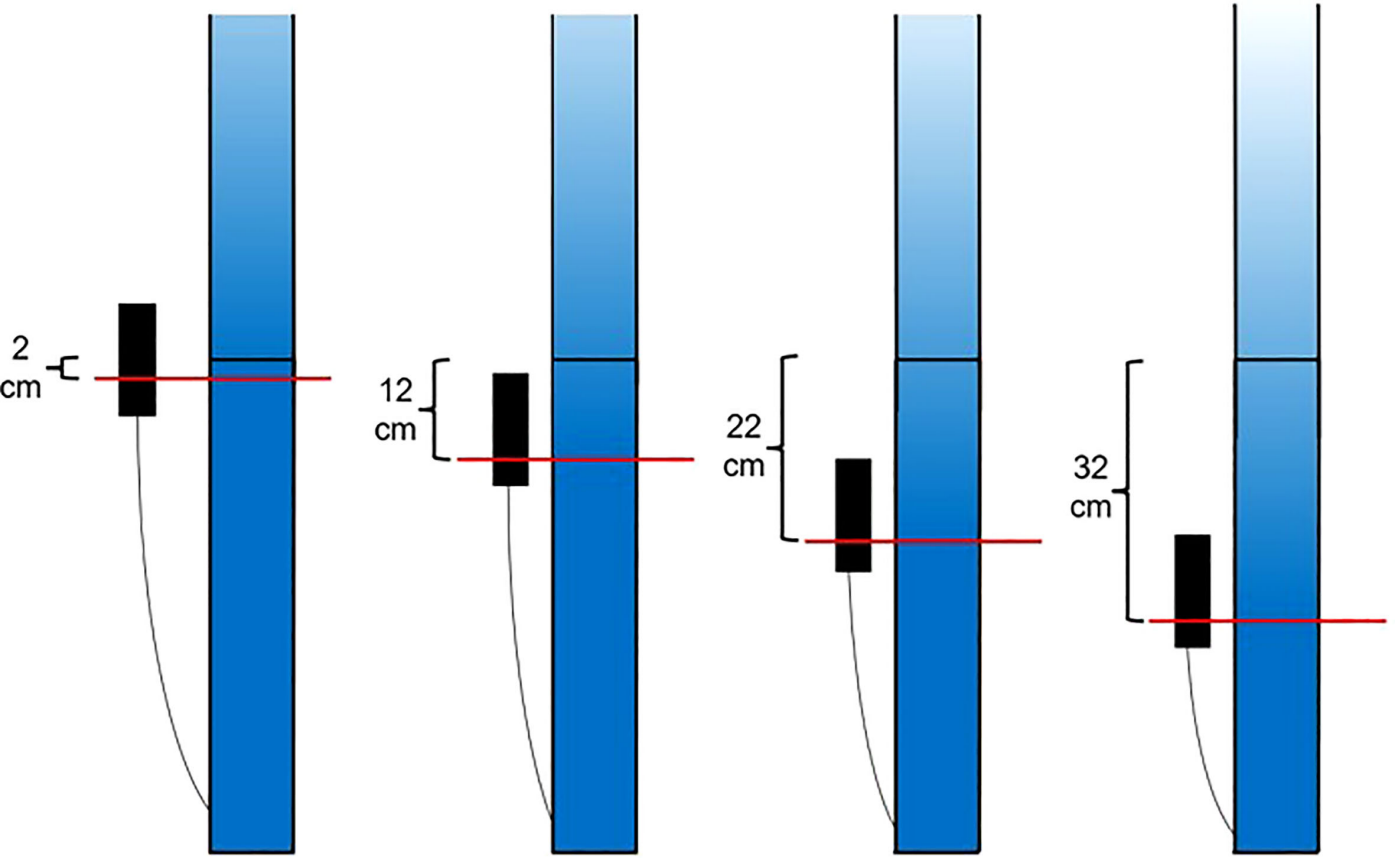
Visual representation of the water table treatments. Alteration of float valve height changes the height of the water table in the wicking bed and consequently, the soil moisture profile in the rooting column.

These water table positions were maintained until harvest (**Figure 5**).

### 2.6 Measurements

Soil moisture measurements were taken weekly using the TDR meter, beginning at 0 DAP. Measurements were taken by touching the TDR meter to the screws on a measurement port and then taking no fewer than three readings, of which the largest 2 measurements had to be no more than 30 µs apart. Measurements continued until this criterion was satisfied. The largest reading in the set was considered the “true” reading for that measurement port on the day of measurement. This protocol was devised to minimize noise within the measurement dataset caused by poor electrical contact between the TDR meter and the screws of each SMM port. Soil moisture measurements were accompanied by water use measurements, taken by reading the water level on the reservoirs via the sight-glass. The water level would be read as the length in cm between the bottom of the meniscus on the sight-glass and a drain hole drilled in the side of the reservoir near the top, which determined the starting water height each time the reservoir was refilled. These changes in water height in the reservoir were converted to volumes by multiplying by the reservoir surface area. Water use measurements were not taken for the first 16 days of the growth cycle, as the rate of water use by the seedlings was not sufficient to lower the water levels in the reservoirs by a measurable amount. Water use was also not counted during a 24-hour period between 26 and 27 DAP, owing to minor adjustments to the float valves to drain excess water from the bodies of the float valves and re-adjust the water table positions, which affected the water reservoir levels. Finally, water use measurements were not taken for a 48-hour period between 31 and 33 DAP owing to the re-equilibration period of the soil moisture profiles following the treatment imposition.

Plants were harvested at maturity, at 86 DAP. Shoots were cut at soil level and pods were counted. For a pod to be counted, a minimum size threshold was set of at least 1cm in length at time of harvest. Plants were placed in a forced-air dryer at a temperature of 60°C for five days. Once dry, Shoot Dry Mass was measured. Pods were then shelled, and seeds were counted and weighed. Plant height was measured and stem width was determined using a caliper at the base of the stem.

To measure the approximate rate of evaporation of water from the system in the absence of plants, soil moisture profiles and reservoir readings were taken five days post-harvest (91 DAP). Soil moisture profiles were comparable to those on the date of harvest and water depletion from the reservoirs was negligible in all pots (data not shown).

Roots were extracted from the pots by removing the polyethylene liner from the PVC pipe, cutting the liner lengthwise and gently separating the soil medium from the roots. Roots were then thoroughly washed to remove soil. Roots were then placed in a forced-air dryer at a temperature of 60°C for three days, then weighed.

### 2.7 Statistical analysis

Statistical Analysis was performed in SAS v. 9.4 (SAS Institute Inc., Cary, NC, United States). Analysis of variance was conducted using PROC MIXED. There were no missing data. The significance threshold α was set at 0.05 for all measured traits, and treatment comparisons were made by comparing LSMeans with protected LSD tests.

## 3 Results

### 3.1 Soil moisture profiles

**Figure 6:**
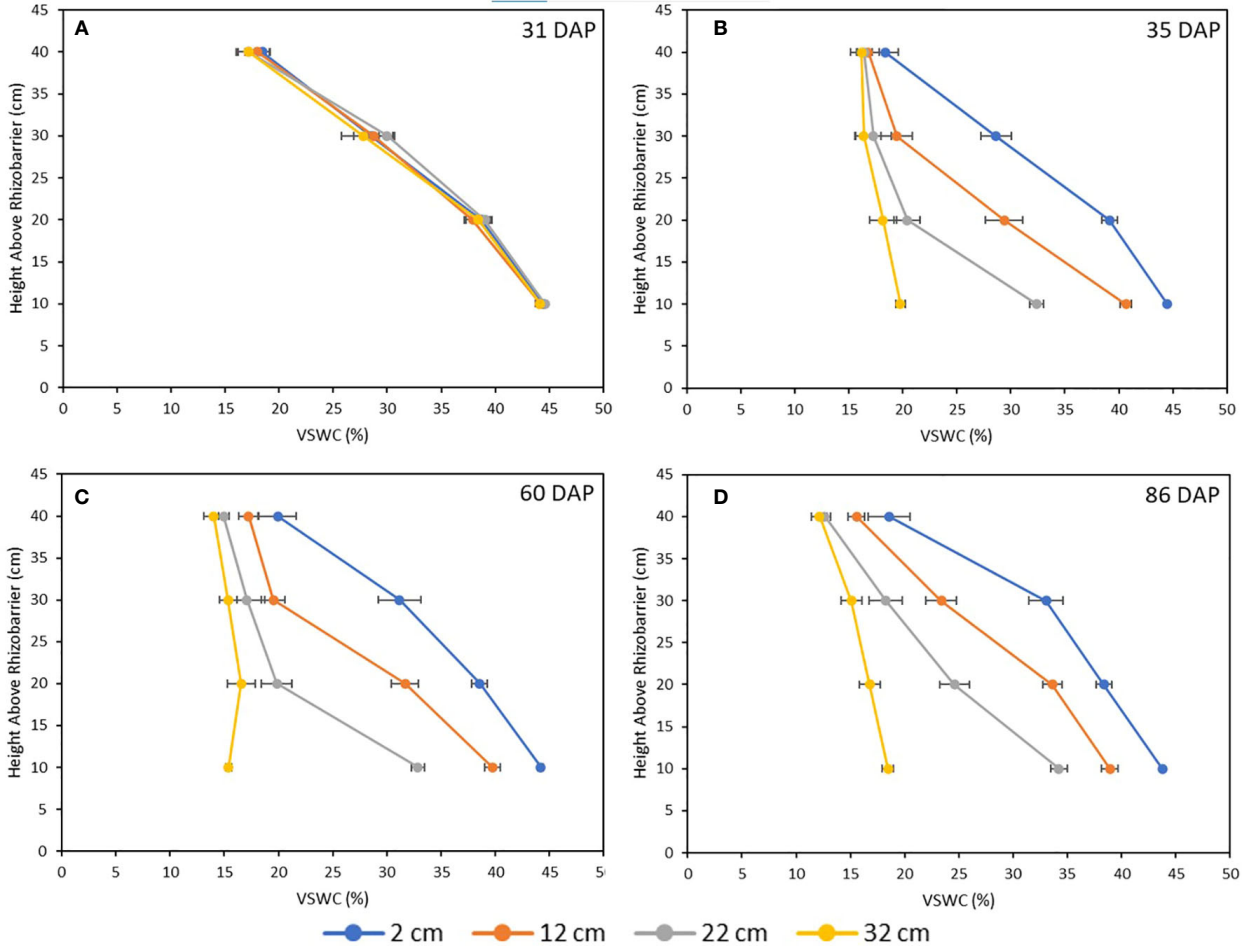
Mean soil moisture profiles for all four drought treatments at 31 DAP (**A**, immediately before stress imposition), 35 DAP (**B**, immediately following re-equilibration period), 60 DAP (**C**, 25 days following re-equilibration) and 86 DAP (**D**, immediately before harvest). Each data point is the mean of five plants, and error bars represent ±1 SE of the mean.

**Figure 7:**
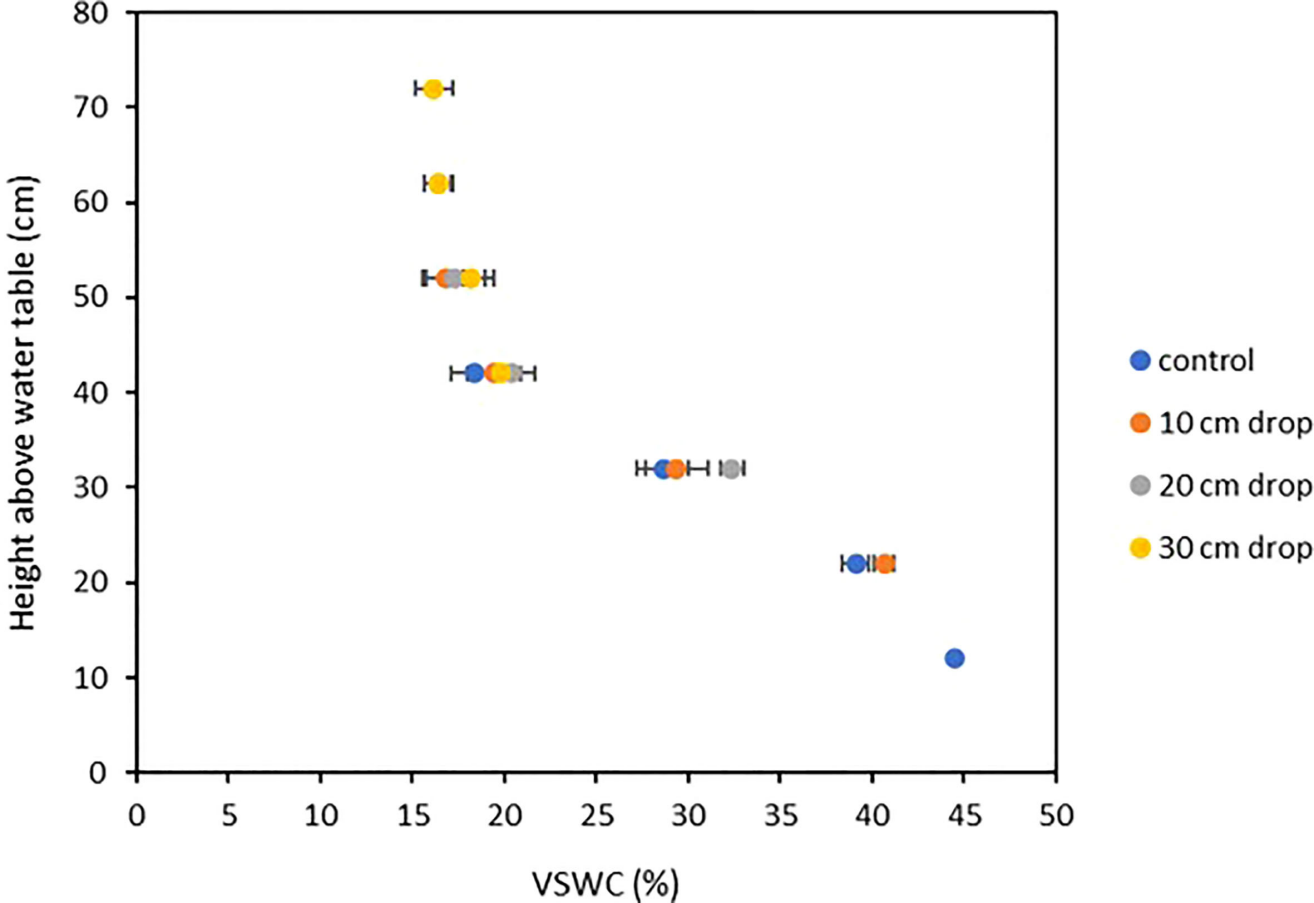
Volumetric-Soil Water Content (VSWC) of various drought treatments compared to TDR port height above water saturation point, four days after treatments were imposed. Each point is the mean of five measurements, made at one soil moisture measurement point in the root column, on five plants from a single stress treatment. Error bars represent ±1 SE of the mean.

Soil moisture profiles were monitored throughout the growth cycle to ensure that VSWC - and therefore the level of drought stress imposed on a plant – was distinct between treatments and remained so from the time of imposition to harvest, as well as to verify that the pre-stress soil moisture levels were comparable between treatments. The soil moisture at 31 DAP, immediately prior to treatment imposition was comparable across all treatments. After water table heights were changed in the stress treatment pots, their measured VSWC dropped rapidly over two days, then stabilized by four days after the adjustment (data not shown). Beginning at 35 DAP, the treatment-mean soil moisture curves were quite stable until harvest (**Figure 6**). Thus, 35 DAP was judged to be the end of the soil moisture re-equilibration period and considered the starting point for post-stress water use measurements. Further, at 35 DAP, the soil moisture curves of the four treatments were clearly distinct from one another, and remained so for the remainder of the experiment (**Figure 6**). **Figure 7** shows treatment-mean VSWC at the four different measurement ports, as a function of height above the water table. All mean soil moisture values across treatments fit neatly along a single soil water release curve, with several measurements from differently placed SMM ports corresponding precisely to one another as a function of height above their respective pot-specific water tables.

### 3.2 Single-plant water use

**Figure 8:**
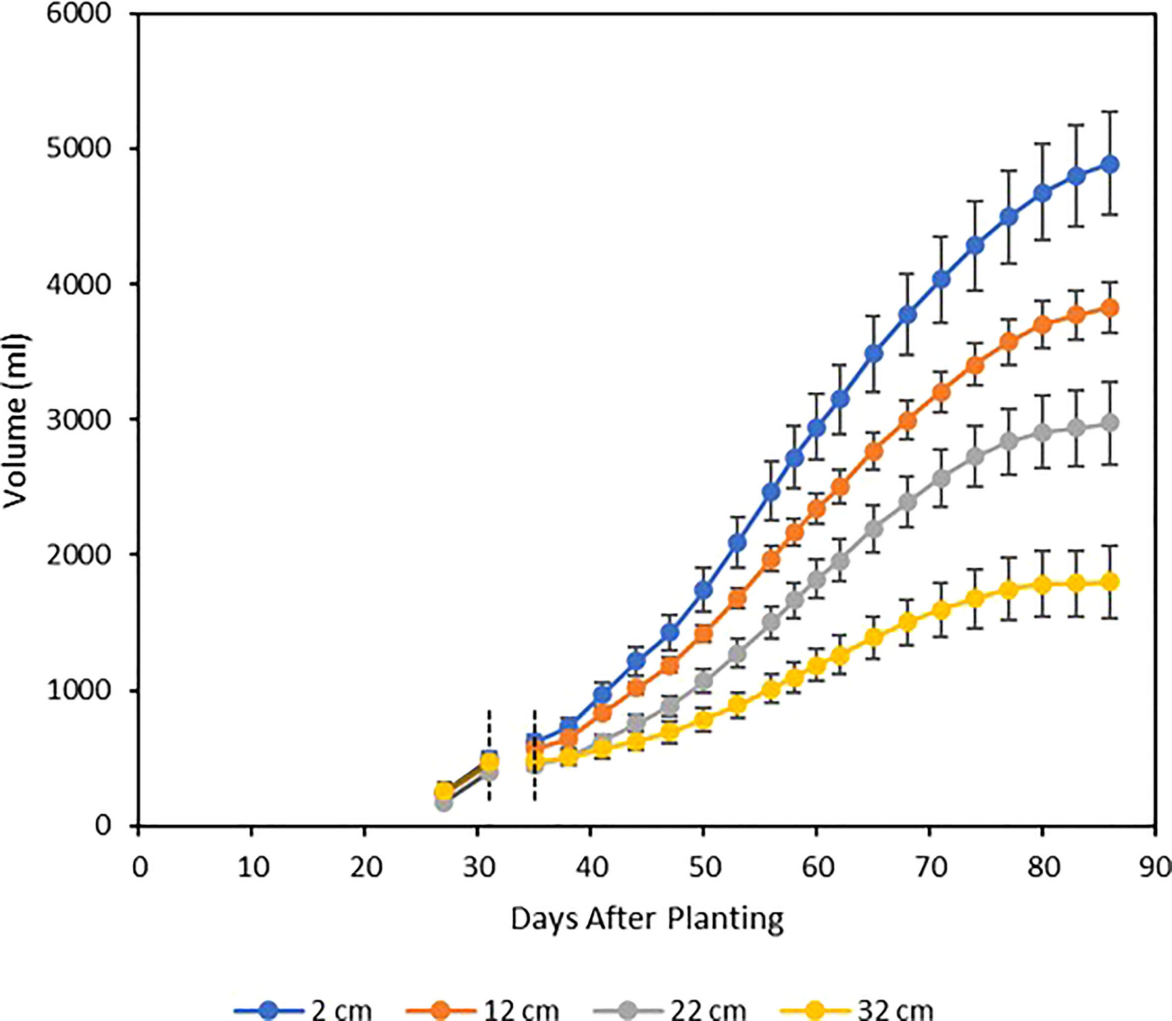
Mean cumulative water use by treatment, beginning four days before imposition of stress and continuing until harvest. The soil moisture re-equilibration period resulted in a four- day gap from 31-35 DAP, during which no valid water use measurements could be taken for statistical analysis. Each curve represents the mean of five plants, and error bars represent ±1 SE of the mean.

Single-plant water use was strongly affected by the soil moisture treatments. During the prestress period from 27 to 31 DAP, water use was not found to vary between treatments, but treatment differences in water use seemed to appear immediately following the four-day re-equilibration after imposition of the treatments, and cumulative water use was clearly differentiated between the treatments from that point until harvest (**Figure 8**). Cumulative water use for each treatment followed a sigmoidal curve, with each treatment reaching maximal rate of water use at roughly 56 DAP, before beginning a slow decrease which continued until harvest. At harvest, cumulative water use showed significant differences between treatments, with these values being statistically distinct in all cases except between the 12 and 22 cm treatments (**Table 1**; **Figure 8**).

**Table 1:** Results of Type-3 analysis of variance for growth, yield and water use traits between drought treatments.

Water Table Height Below Rhizobarrier	Shoot Dry Weight (g)	Root Dry Weight (g)	Pod Number	Seed Number	Yield (g)	Plant Height (cm)	Stem Width (mm)	Hundred-Seed Weight (g)	Seeds Per Pod	Pod Ratio	Harvest Index	Root/Shoot Ratio	Cumulative Water Use (ml)	WUE (g/l)	Seed WUE (g/l)
**2 cm**	14.04 a	2.00	26.6 a	50.6 a	6.80 a	76.8a	4.13a	13.67	1.90	1.89	0.489	0.14a	4397 a	3.21 a	1.57 a
**12 cm**	11.46 b	2.31	19.8 b	38.0 b	5.49 b	72.4a	4.05a	14.44	1.95	1.73	0.481	0.20b	3360 b	3.43 a	1.65 a
**22 cm**	8.59 c	1.74	13.6 c	29.0 c	4.57 c	52.8b	3.61b	15.88	2.19	1.58	0.537	0.20b	2576 b	3.38 a	1.82 a
**32 cm**	6.29 d	2.04	12.2 c	23.2 c	3.13 d	46.1b	3.27 b	13.67	1.98	1.93	0.498	0.33c	1332 c	5.11 b	2.51 b
**Standard Error**	0.7	0.24	1.6	2.4	0.3	4.5	0.12	0.85	0.2	0.11	0.0218	0.02	263	0.35	0.17
**Significance**	***	ns	***	***	***	***	***	ns	ns	ns	ns	***	***	**	**

Levels of significance for treatment effects shown for p< 0.01 (**) and p< 0.001 (***). Values within a column which are not significantly different from one another according to a protected LSD test at α = 0.05 are denoted with the same letter.

ns, not significant.

### 3.3 Harvest traits

**Figure 9:**
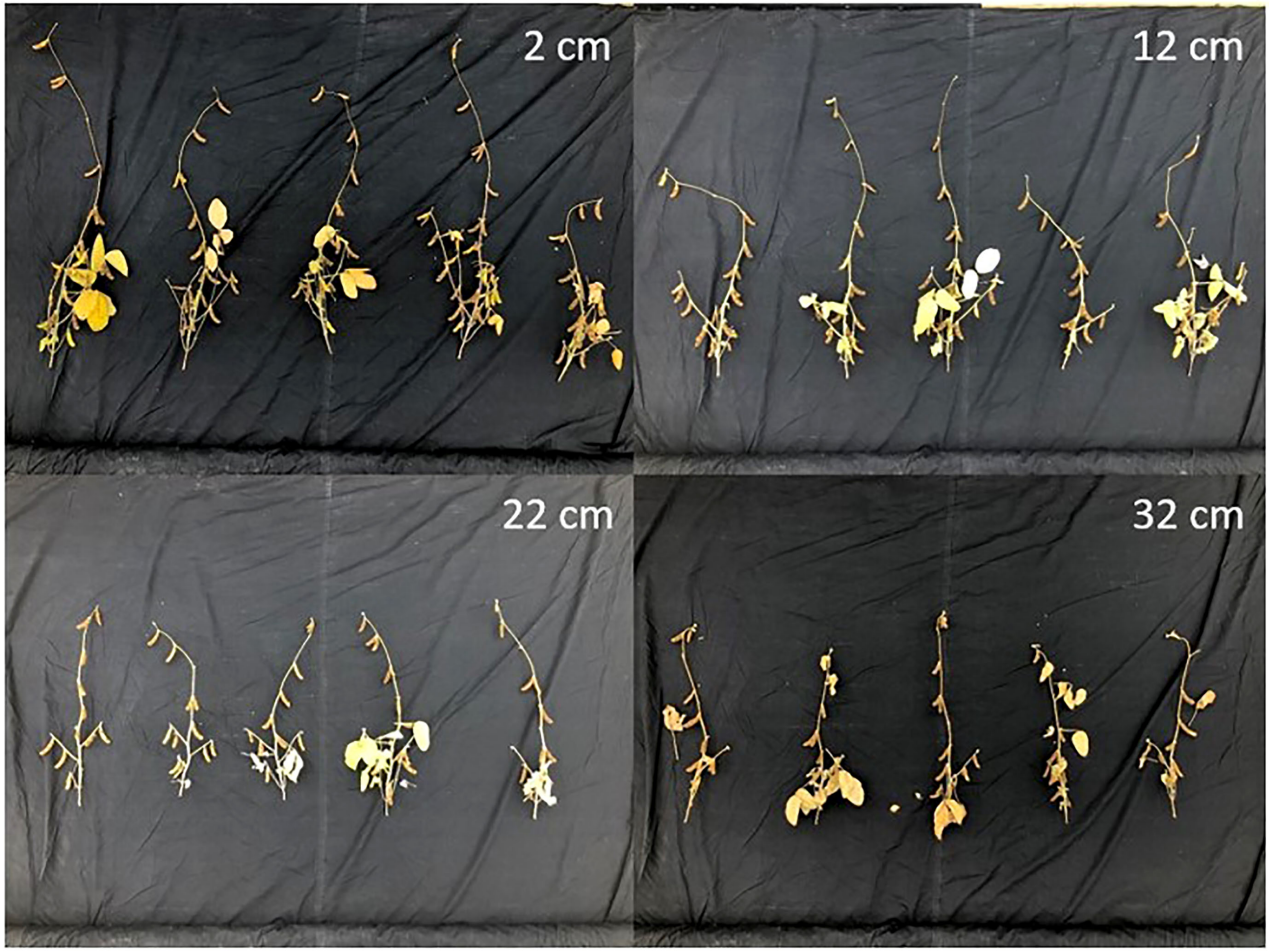
Shoot harvest images for each plant within a treatment group.

Results of the analysis of variance can be seen in **Table 1**. There was significant variation between treatments for several soybean growth and yield traits. Significant treatment effects were observed for shoot dry weight, pod number, seed number, seed yield, plant height, stem width, root/shoot ratio, water use efficiency (WUE; shoot dry weight/cumulative water use) and seed-based water use efficiency (sWUE; seed dry weight/cumulative water use) all of which were significant at p<0.001, except for WUE and sWUE, which were significant at p<0.01. Treatment effects were not significant with respect to root dry weight, hundred-seed weight, seeds per pod, or pod ratio (pod number/shoot dry weight) or harvest index (HI; seed yield/shoot dry weight). Additionally, differences between treatments in the size of the shoots were readily apparent with visual observation (**Figure 9**).

Means comparisons were also conducted. For shoot dry weight and yield, treatment means were all significantly different from one another, indicating four distinct levels of water stress. For pod number and seed number, treatment means were all significantly different from one another, except between the 22 cm and 32 cm treatments. For root/shoot ratio, treatment means were all significantly different from one another, except between the 12 and 22 cm treatments. Water use efficiency and seed-based water use efficiency, were both significantly higher in the 32 cm treatment than any of the other treatments; the other three treatments were not significantly different from one another. Full means comparisons can be seen in **Table 1**.

## 4 Discussion

### 4.1 Comparison to field trials and gravimetric methods

Plant water use measurement techniques at present are expensive, labor-intensive, time consuming, complex or confounded by several factors such as soil evaporation and plant fresh weight accumulation, which affect the accuracy of gravimetric measurements. This study aimed to demonstrate the functionality of a bottom-watered, mineral soil-based plant system that could impose varying levels of soil water deficit stress, and be easily scalable for high-throughput phenotyping, including measurement of water use traits. The system presented in this work allows for soybean growth to maturity in a controlled environment, under a selected and well controlled level of water availability, which significantly impacts growth and water use, as well as final yield and yield components. Once the soil moisture levels are set and equilibrated within the soil columns, the system works with minimal intervention, requiring only intermittent replenishment of water in the reservoirs and the direct reading of the water level on the reservoir sight-glasses for water use calculations. Plants remained healthy throughout the growth cycle and were able to produce seed at maturity.

For effective characterization of soybean drought response using this system, the effects of soil moisture profile change on growth and yield traits should mimic those seen during drought in field environments. When drought stress is induced around the R1 growth stage (~31 DAP) and over the entire growth cycle, shoot dry weight and pod number are reduced (Kpoghomou et al., 1990; Visser, 2014; Wei et al., 2018). In the present study, water stress also induced a significant reduction in seed number and yield. Similar yield or biomass component reductions as a result of water deficit stress are also frequently observed in both field and outdoor-potted settings (Brown et al., 1985; Sloane et al., 1990; Araya et al., 2010; Visser, 2014; He et al., 2017).

The present study did not, however, identify any significant changes in root dry weight, 100-seed weight, seeds per pod, pod ratio or harvest index in response to drought stress. In a multi-year field study of multiple varieties, including *OAC Drayton*, 100-seed weight, seeds per pod and harvest index were also not found to vary significantly with drought stress (Visser, 2014). The effects of drought stress on root dry weight and 100-seed weight in other works, though often significant, were sometimes modest compared to the reductions observed in yield or seed number (Brown et al., 1985; Sloane et al., 1990; Fried et al., 2019). Further, changes in both the 100-seed weight and seeds per pod in response to drought may vary substantially with cultivar (Visser, 2014; He et al., 2017). Some works were not consistently able to identify significant reduction in root dry weight under drought conditions (Guttikonda et al., 2014). Finally, the root/shoot ratio – which was found to vary significantly in the present experiment, is calculated based on the root dry mass and the shoot dry mass. As the root dry mass was not found to vary substantially, the differences in root/shoot ratio are largely attributable to the variation in shoot dry mass across treatments.

As accurate cumulative water use and WUE calculations require direct measurement of plant water use, direct comparisons between the present work and the behavior of soybean in a field setting with respect to these water use traits is difficult. In the field, it is challenging to differentiate between plant water use via transpiration, and other losses of water from the system such as evaporation and percolation below the root zone. From comparisons with other controlled environment studies, the cumulative water use curves obtained from the present experiment bear a similar sigmoidal shape to the cumulative water use curves obtained using gravimetric methods in previous pot experiments in which soybean was grown to physiological maturity (Gebre and Earl, 2021). The overall curve shape over the complete developmental cycle, in combination with the i) near constant soil moisture profiles within the pots, and ii) the negligible post-harvest reservoir depletions, demonstrate that nearly all of the water from the reservoirs was consumed by the plants, as opposed to being lost to evaporation or associated with changes in VSWC in the pots. The low soil surface evaporation losses may be attributable to the bottom-watering mechanism, which allows for the top layers of the soil to remain relatively dry. This reduces the soil water’s exposure to open air, greatly limiting the opportunity for evaporation compared to top-watered culture systems. Overall, the phenotypic responses obtained from the current experiment closely reflect the observations made of soybean response to drought stress in field environments, as well as water use traits in other controlled-environment settings.

### 4.2 Comparison to related pot culture systems

Many systems currently exist which supply water to plants by means of capillary irrigation (Semananda et al., 2018). Similar bottom-watered drought-stress control systems to the one shown here have been described previously (Haan and Barfield, 1971; Snow and Tingey, 1985; Wookey et al., 1991; Saulescu et al., 1995; Fernández and Reynolds, 2000; Araya et al., 2010; Couso et al., 2010; Couso and Fernández, 2012; Marchin et al., 2020). Early versions of these systems relied on a moisture-conducting porous material, above which the growth medium would be placed (Haan and Barfield, 1971; Snow and Tingey, 1985). The water table would be adjusted to different heights within the porous material, resulting in differing moisture levels at the interface between the conductive medium and the growth medium. These methods did not, however, attempt to produce a change in the soil moisture profile throughout the depth of the growth medium itself. The mechanism for drought stress in these systems (ie. varying water stress via a two-dimensional absorbing surface) does not reflect the reality of soil moisture *in situ*, where soil moisture varies across soil depth. Namely, plants often draw water from different depths depending on growth stage (Guo et al., 2016; Gebre et al., 2022). Some later iterations of the system made improvements to the original mechanisms proposed (Wookey et al., 1991; Fernández and Reynolds, 2000), however none of these modifications diverged from the original concept of a 2-dimensional absorption interface between the porous, water-conducting medium and the growth medium. Some systems developed independently of these techniques attempted to induce varying levels of drought stress using upward wicking of water through a soil column, however none of these systems were able to maintain a constant soil moisture profile over prolonged periods throughout the depth of the rooting columns (Iseki et al., 2018; Santos et al., 2020).

The system presented above improves on these techniques by providing a consistent yet adjustable soil-moisture profile through the depth of the entire rooting column itself by taking advantage of the unique hydrological properties of a specialized soil medium, identified by Cichello (2023). The results in Cichello (2023), along with those reported here, broadly support the hypothesis that lowering the pot-specific water tables functions to lower the soil water release curve downwards by a corresponding height. The results from the soil moisture measurements before, during and after the growth cycle affirm that the soil medium remains capable of maintaining the desired soil moisture profile by wicking water upwards to replace water drawn from the soil column (such as through root uptake), with relatively little sensitivity to the position of the soil water release curve in the soil column.

The present study also expands on prior work with similar systems by measuring daily or cumulative single-plant water use and measuring growth and yield traits in an agronomically important species, grown to maturity. Previous studies have focused on measuring factors relating to leaf-level physiology (Snow and Tingey, 1985; Wookey et al., 1991; Fernández and Reynolds, 2000; Marchin et al., 2020). None of these studies attempted to directly measure bulk water flow out of the reservoir. Additionally, while some of the previous works have measured some whole plant traits such as biomass (Wookey et al., 1991; Fernández and Reynolds, 2000; Marchin et al., 2020), none have measured a full suite of growth and yield traits, particularly in an intensely cultivated row crop such as soybean.

### 4.3 Further optimization of system configuration

The capacity to directly measure water use in a high-throughput fashion is a valuable property for a drought tolerance trait phenotyping system. This system can be adapted such that a single float valve can be connected to a manifold which distributes water to several wicking beds in parallel. Such an arrangement precludes measurement of water use of single plants, but greatly simplifies the construction of the apparatus in the case where hundreds of entries are to be compared for traits such as biomass, yield and yield components. We are unaware of any similar works that attempt to apply a bottom-watered, soil-moisture-based system to these types of phenotyping efforts. Our system, with the ease and consistency of treatment application over a potentially large number of pots, should be well suited to such applications.

The current configuration of the culture system could also be optimized in the future to reduce design inefficiencies. The lower wicking bed of the system has a height of 60 cm. This height was chosen in part because it was originally hypothesized that the soil mix used in the pots would require large drops in the water table to yield a decrease in rooting column soil moisture sufficient to induce physiologically significant levels of drought stress. The current study demonstrates that the soil moisture profile of the soil medium used is, in fact, quite sensitive to even small reductions in the water table height (**Figure 6**), with a drop of as little as 10 cm inducing a statistically significant decrease in many measured traits, and only a 30-cm drop required to produce the extreme responses observed for the 32 cm treatment. Therefore, at least 20 cm of the height of the wicking bed may be redundant to produce the drought responses observed here in soybean. Reducing the wicking bed height and therefore the overall size of the pot units of the culture system will reduce the cost and workload of recreating this system, as less soil will need to be prepared, and will also reduce the mechanical load on the structure supporting the culture system, which is an important logistical consideration for studies where many genotypes must be tested and many pots prepared.

Reduction in height of the rooting column may also benefit plant growth and system efficiency, as root biomass tends to be more heavily distributed in the uppermost soil layers (Gebre and Earl, 2020), which in the current experiment tended to display the lowest VSWC (**Figure 6**). The distribution of roots in the uppermost, driest soil layers in this system may result in a baseline drought effect experienced by all plants, including those in the 2 cm treatment. The cumulative water use values and single plant dry weights obtained in Gebre and Earl (2021) are considerably higher (Water Use = 39400 ml ± 4600, SDM = 57.1 g ± 5.1 for the plants in their water-replete treatment) than those obtained in the present study. Reduction of the rooting column height could increase the VSWC in the upper soil layers from ~20% to ~30% by lowering the height of the planted seed by 10 cm along the soil water release curve shown in **Figure 7**, thus alleviating any initial drought stress experienced by the plants. Other factors may also have contributed to the lower growth and water use in this study compared to Gebre and Earl (2020); namely, differing cultivars, the different soil media used and, especially, the different quality and quantity of photosynthetically active radiation (sunlight supplemented with metal halide and high-pressure sodium lamps in a greenhouse in that study; LED lamps in the current work).

Finally, pots in this experiment were lined with polyethylene sleeves to facilitate removal of the root systems for harvest, but the sleeves were not included for the border pots. Unexpectedly, plants in the border pots grew poorly. Subsequent experimentation with the system confirmed that omission of the polyethylene sleeves resulted in stunted plants and shallow root growth (**Supplementary Data**). We considered the possibility that the smaller border plants would have resulted in reduced light competition and therefore enhanced growth of plants adjacent to the borders. However, we verified that entirely excluding the border-adjacent pots from the analysis did not fundamentally change the results in **Table 1**. Nevertheless, results presented in the **Supplementary Data** still clearly indicate that the polyethylene liners are necessary for robust plant growth in the system as currently configured.

## 5 Conclusion

The bottom-watered, constant soil moisture drought culture system presented in the current work improves plant drought phenotyping in two ways. First, this system can accurately and precisely apply an easily controllable soil moisture profile to a 50-cm rooting column without the need to periodically replenish water to the potting medium through manual or even automated means. The effects of the altered soil moisture profiles on plant growth and yield traits were observed to be statistically significant. Secondly, this system offers a non-gravimetric, high-throughput method to directly measure single-plant water use in a rooting column while feeding water to the plant through the bottom of the pot, thus minimizing water lost to soil surface evaporation relative to most current methods. The capability of this culture system to set the desired drought level and simultaneously provide a measure of plant water use further increases the utility of the system for large studies. The drought culture system shown here greatly expands the capacity of future research to screen large numbers of plant genotypes for drought response at low cost and with minimal intervention, thereby increasing the statistical power of studies requiring such reliable phenotyping, such as GWAS.

## Data availability statement

The original contributions presented in the study are included in the article/**Supplementary Material**. Further inquiries can be directed to the corresponding author.

## Author contributions

AC designed and carried out both the main experiment and the experiment described in the **Supplementary Materials**, as well as collected all data presented here. AC and HE performed the statistical analysis for the main experiment. AC performed the statistical analysis for the **Supplementary Experiment**. AC and HE both contributed to the writing of the manuscript. All authors contributed to the article and approved the submitted version.
