# Supplementary figures and images for: A novel method for irrigating plants, tracking water use, and imposing water deficits in controlled environments
Source: Front Plant Sci. 2023 Aug 29;14:1201102. doi: 10.3389/fpls.2023.1201102 (PMC10497755; doi:10.3389/fpls.2023.1201102)

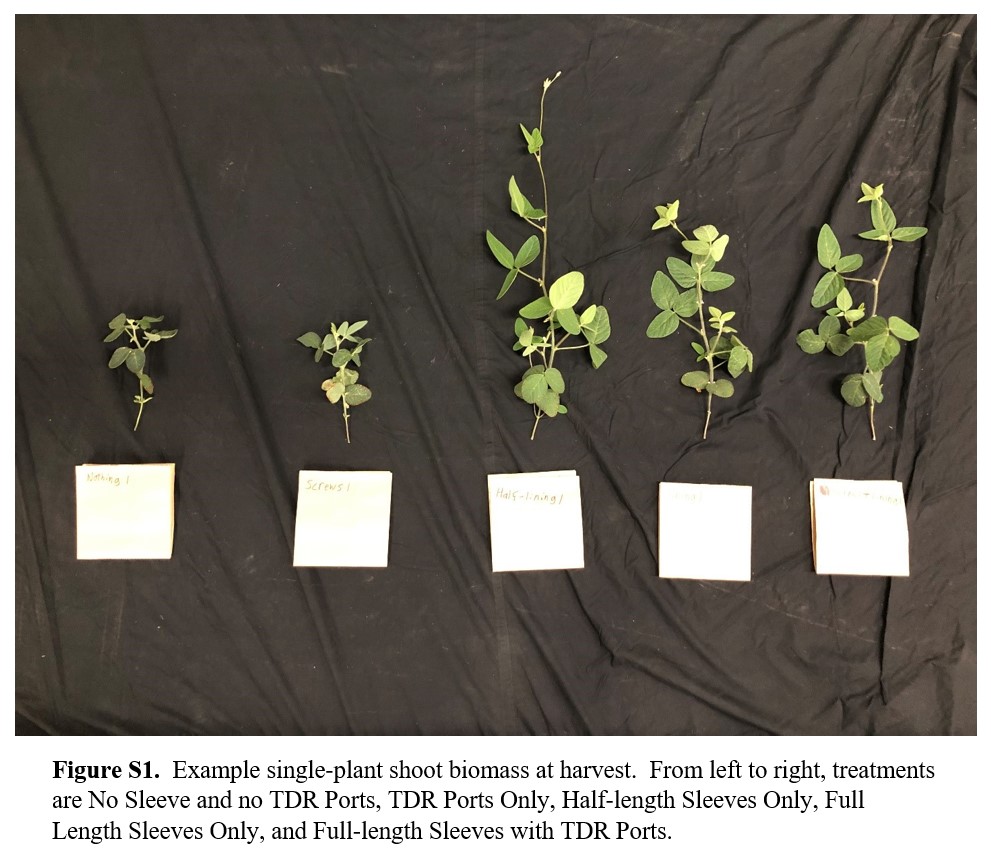

Supplement: Supplementary file 2 [file Image_1.jpg]

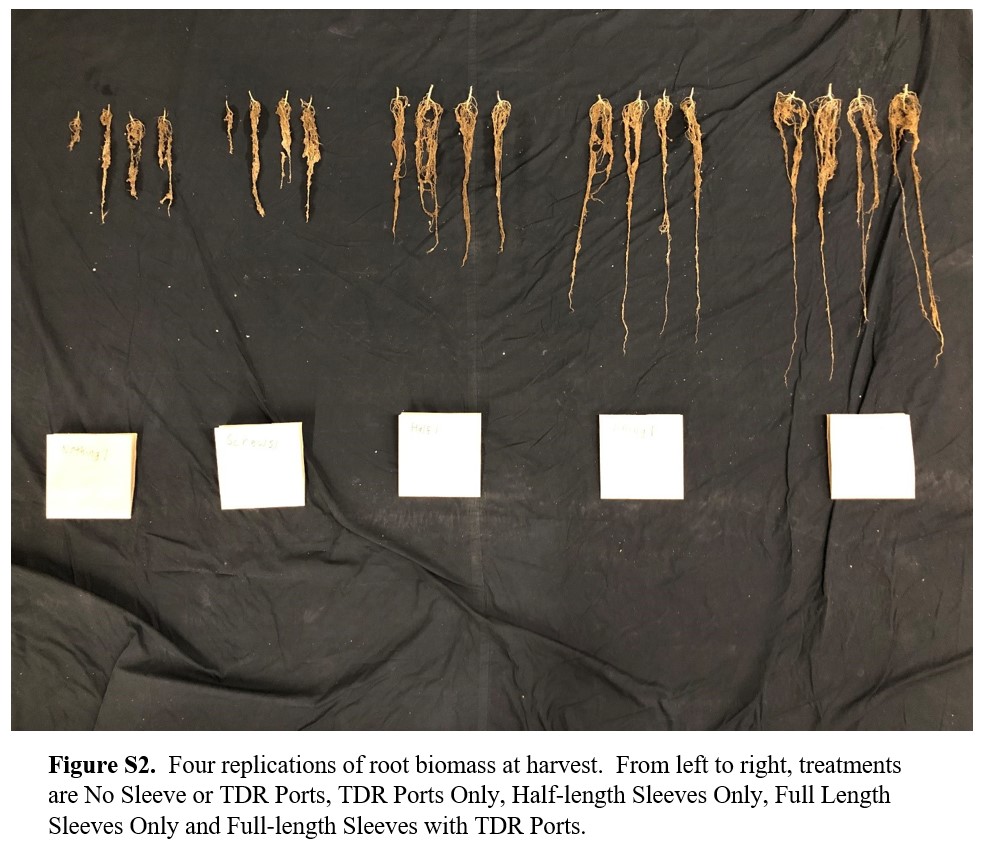

Supplement: Supplementary file 3 [file Image_2.jpg]

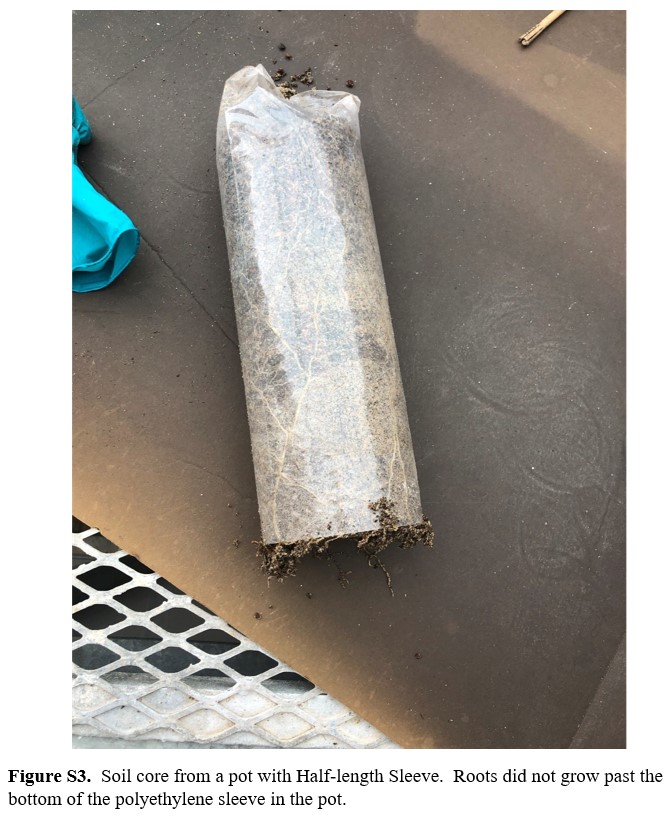

Supplement: Supplementary file 4 [file Image_3.jpg]
