# Supplementary material for: A novel method for irrigating plants, tracking water use, and imposing water deficits in controlled environments
Source: Front Plant Sci. 2023 Aug 29;14:1201102. doi: 10.3389/fpls.2023.1201102 (PMC10497755; doi:10.3389/fpls.2023.1201102)
Supplement: Supplementary file 5 [file Table_1.docx]

***Supplementary Material***

**A novel method for irrigating plants, tracking water use and imposing water deficits in controlled environments**

**Alex Cichello, Austin Bruch, Hugh J. Earl***

**Correspondence:**

**Hugh J. Earl**

**hjearl@uoguelph.ca**

**Objective**

The objective of the experiment described here was to characterize the effect of the rooting column configuration on soybean growth in the bottom-watered system designed to impose drought treatments and measure plant water use. Two potential sources of variation were identified – the stainless-steel screws which facilitate the TDR measurements for soil moisture determination and the polyethylene sleeve lining the inside of the pots.  The lining was originally included in the system to prevent water escape from the TDR access holes (before the screws were adopted as permanent TDR probes), and to facilitate easy extraction of the intact root systems.  All possible configurations were assessed here, with an additional group comprising pots with only polyethylene liners that reach halfway down the depth of the pot.  If the source of the variation in plant growth was, in fact, the sleeve lining, this group would provide stronger evidence to that effect, as well as provide a qualitative measure of how closely tied rooting depth is to sleeve length.

**S2 Methods**

The growth environment was the same as described for the initial experiment.  The growth system was also the same, with a few notable exceptions.  Here, the water supply to the flow ports at the bottom of each pot was not provided by a pot-specific reservoir controlled by a pot-specific float valve.  Instead, we connected an entire rack to one large reservoir via a manifold composed of 3.2 cm ABS pipe, capped at both ends with a 3.2 cm ABS plastic cap, with a port installed into one of the caps to supply water from the float valve.  Installed in each manifold was also a hose barb to connect a sightglass for the entire manifold and thus, each pot connected to that manifold.  The manifold rested under the wooden frame of the growth system.  As this configuration made the sight glasses on each pot redundant, the wicking bed connections otherwise used for sightglasses was instead used to connect pots together in a daisy-chained fashion.  This arrangement both eliminated unnecessary clutter caused by redundant sightglasses and facilitated more efficient flow of water between pots in a rack.  Instead of the water control level acting as a treatment, control levels were maintained at 2 cm below the rhizobarrier and rooting column configuration was the experimental treatment.

**S2.1 Pot Preparation**

4 replications of each of the following pot configuration treatments were prepared:

-PVC rooting column only

-PVC rooting column, with 8 stainless steel screws installed in four pairs, at 10 cm height intervals as would be installed to measure soil moisture via TDR.

-PVC rooting column, with a polyethylene sleeve lining the inside of the rooting column to a depth of ~25 cm below the soil surface.

-PVC rooting column, with a polyethylene sleeve lining the entire inside of the rooting column.

-PVC rooting column, with 8 stainless steel screws installed in four pairs, at 10 cm height intervals as would be installed to measure soil moisture via TDR, and a polyethylene sleeve lining the entire inside of the rooting column.

**S2.2 Experimental Design**

Here two racks were prepared, each comprising one row of 10 test pots (plus two border plants per rack).  Each pot within a rack was connected to a single manifold.  This minimized between-treatment variation of soil moisture profiles by ensuring that the wicking bed water level in each pot within a rack was held at the same control level.  Plants were grown on two such racks, on adjacent benches in the growth room.  Pots were arranged such that each rack had exactly two pots of each treatment but treatments were otherwise randomly distributed.  Despite this pot layout, variation between the two racks was expected to be minimal and thus a Completely Random Design (CRD) was adopted for statistical analysis.

Seeds of *OAC Drayton* were planted 3 per pot and thinned to 1 per pot upon emergence.  Orange plastic test caps were placed on pots until emergence to minimize evaporation, however no white gravel was used to control evaporation after this point, as qualitative observations from previous experiments found the effect of the gravel to be negligible with respect to the prevention of soil evaporation.  Plants were given the same fertilization regimen as in the initial experiment.  Fly paper was used to control pests, in combination with insecticide application (Beleaf, FMC Canada, Mississauga, ON, Canada) which was applied to the plants at 27 DAP.

Shoots were harvested at 43 DAP and photographed.  Shoots were then placed in an 80ºC forced-air dryer for at least 3 days and subsequently weighed.  Roots were carefully removed from the growth medium at 44 DAP, then thoroughly washed and photographed.

Analysis of Variance was performed on shoot dry weight samples using PROC Mixed in SAS v.9.4 (SAS Institute, Carey, NC, USA).

**S3 Results**

**S3.1 Shoot Dry Mass**

Shoot dry mass varied significantly with treatment (Table S1).  Means comparisons revealed only two significantly different classes at α = 0.05.  Each of the five treatment means segregated perfectly into one of these two classes.  The two classes can be characterized as pots with polyethylene sleeves and pots without polyethylene sleeves.  This result represents strong evidence that the absence of polyethylene sleeves strongly inhibits plant growth in this culture system.

Still further evidence of this dependence can be seen qualitatively by examining photographs of shoots and roots.  Figure S1 shows clear visual differences among shoots from the various treatments, especially between the two classes identified by means comparisons. Figure S2 shows clear visual differences among roots between treatments and low variation within treatments.  In contrast, no other clear differences can be seen associated with the presence or absence of TDR ports, or with respect to an interaction between the sleeves and the ports.

The difficulty in avoiding root breakage while extracting the root biomass from the rooting columns and thoroughly washing the root biomass precluded any valid root dry mass measurements.  It is noteworthy that in the half-lined pots, the roots did not grow past the end of the sleeves and therefore only reached roughly halfway down the rooting column (Figure S3).  The exact mechanism responsible for the growth responses to sleeve presence or absence, whether chemical or mechanical, remains unclear.

| Treatment | Shoot Dry Weight (g) |
| --- | --- |
| No Sleeves or TDR Ports | 0.97a |
| TDR Ports Only | 1.10a |
| Half-length Sleeves Only | 2.99b |
| Full-length Sleeves Only | 2.79b |
| Full-length Sleeves and TDR Ports | 3.47b |
| Standard Error | 0.26 |
| Significance | *** |

**Table S1.** Results of type-3 analysis of variance for shoot dry weight for each liner treatment. *** denotes a p-value of less than 0.001 for the treatment effect. Values within a column which are not significantly different from one another at α = 0.05 according to a protected LSD test are denoted with the same letter.
